# Heteroepitaxial Growth of α‑Ga_2_O_3_ by MOCVD on *a*‑, *m*‑, *r*‑, and *c*‑Planes of Sapphire

**DOI:** 10.1021/acs.cgd.5c00183

**Published:** 2025-07-29

**Authors:** Khai D. Ngo, Indraneel Sanyal, Matthew D. Smith, Martin Kuball

**Affiliations:** Centre for Device Thermography, 1980 University of Bristol, Bristol BS8 1TL, U.K.

## Abstract

Ga_2_O_3_ thin films were deposited simultaneously on (112̅0) *a*-plane, (101̅0) *m*-plane, (0001) *c*-plane, and (011̅2) *r*-plane sapphire substrates using metal–organic chemical vapor deposition (MOCVD) and characterized by X-ray diffraction (XRD) and atomic force microscopy (AFM). The different surface energy and strain conditions imposed by each sapphire plane make the choice of substrate orientation critical to the stabilization of the α-phase. β-Ga_2_O_3_ nucleation was found to be preferential over α-Ga_2_O_3_ on sapphire orientations with <11̅00> α-Al_2_O_3_ present (c- and *a*-planes) when grown under the same conditions. In contrast, α-Ga_2_O_3_ is preferred during the initial stages of growth on the *r*- and *m*-plane, although suppression of island growth is required to prevent the formation of inclined facets on which β-Ga_2_O_3_ might nucleate. Transmission electron microscopy (TEM) provided a direct confirmation of this growth for *r*-plane substrates. Classical nucleation theory was applied to rationalize these observations and guide the search for the growth window of α-Ga_2_O_3_. As a result, decreasing the VI/III ratio and increasing the TEGa flow rate were found to be effective in realizing phase-pure α-Ga_2_O_3_ on *a*-plane sapphire by MOCVD with good structural quality (62 arcsec full width half-maxima of X-ray rocking curve), though the equivalent growth on *c*-plane substrates yielded mixed-phase β- and κ-Ga_2_O_3_—another metastable phase of Ga_2_O_3_, instead. Growth on the *m*-plane resulted in the smoothest surface morphology and thickest phase-pure α-Ga_2_O_3_ film, indicating that it is the most promising substrate orientation for future device manufacturing.

## Graphical abstract

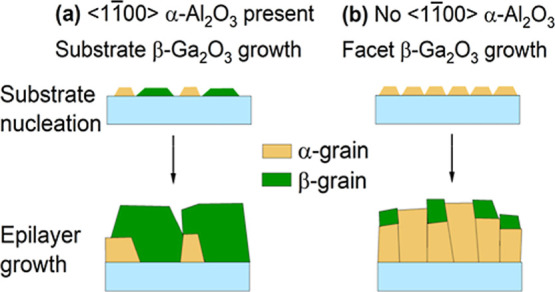

## Introduction

Corundum α-Ga_2_O_3_ (space group *R*3̅*c*) boasts the widest bandgap of all Ga_2_O_3_ polymorphs (5.1–5.3 eV)^1^ and is isostructural with α-Al_2_O_3_, commonly known as sapphire. This permits not only the growth of high-quality domain-matched α-Ga_2_O_3_ layers on economical substrates^2^ (sapphire being available at significantly lower cost than native Ga_2_O_3_ substrates used for high-quality β-Ga_2_O_3_ growth) but also extensive bandgap engineering by alloying with Al over the full composition range from *x* = 0 to 1 in α-(Al_*x*_Ga_1–*x*_)_2_O_3_, leading to a bandgap range of 5.1–8.8 eV.^3–5^ Such compositional control is generally more difficult in the monoclinic phase, where β-(Al_*x*_Ga_1–*x*_)_2_O_3_ films typically suffer from local segregation of Al and Ga (e.g., on (001) and (2̅01) β-Ga_2_O_3_ substrates)^6,7^ or phase segregation of β- and γ-Ga_2_O_3_ (e.g., on (010) β-Ga_2_O_3_ substrates)^8,9^ toward high Al content. Up to 99% Al composition β-(AlGa)_2_O_3_ films have been achieved on (100) β-Ga_2_O_3_ substrates, but the film quality degrades with increasing Al content.^7^ In contrast, α-(AlGa)_2_O_3_ films do not degrade or even improve in crystallinity toward Al-rich regimes.^4,5^ Finally, it is worth highlighting that, similar to β-Ga_2_O_3_, n-type conductivity of α-Ga_2_O_3_ films can be achieved by Si,^10–14^ Sn,^15,16^ or Ge doping,^16,17^ with controllable carrier density over the range of 10^17^–10^19^ cm^–3^ and electron mobility up to 98.7 cm^2^ V^–1^ s^–1^ having been reported.^14^ These qualities make α-Ga_2_O_3_ an attractive material for the fabrication of high-breakdown power devices like Schottky diodes,^10,11,17^ field-effect transistors,^13,14^ and optoelectronic devices such as solar blind photodetectors.^18^

Despite these advantages, the growth of α-Ga_2_O_3_ is challenging because of its metastability. Thin films of phase-pure α-Ga_2_O_3_ can transform into the most thermodynamically stable polymorph β-Ga_2_O_3_, typically at temperatures above 600–650 °C.^19–21^ Up to now, mist chemical vapor deposition (mist-CVD)^2,13,22,23^ and halide vapor phase epitaxy (HVPE)^24–26^ have proven to be the most effective methods capable of realizing thick layers of α-Ga_2_O_3_ on *c*-plane sapphire; this might be due to the presence of HCl that acts as a catalyst and aids the formation of α-Ga_2_O_3_.^27^ For epitaxial techniques without HCl such as pulsed laser deposition (PLD), molecular beam epitaxy (MBE), or standard metal organic chemical vapor deposition (MOCVD), Schewski et al. found that only a pseudomorphic interlayer of α-Ga_2_O_3_ could be stabilized on *c*-plane sapphire before transitioning to (2̅01) β-Ga_2_O_3_ as soon as the film relaxes.^28^ Although only a few monolayers of α-Ga_2_O_3_ were stabilized, this study demonstrated that substrate-imposed strain conditions are a key factor in the stabilization of α-Ga_2_O_3_. A later study by Schowalter et al. showed that the thin pseudomorphic interlayer is actually α-(AlGa)_2_O_3_ with ∼65% Al, rather than pure α-Ga_2_O_3_ as previously assumed, suggesting that Ga–Al interdiffusion between the film and substrate may occur during its formation.^29^ Nonetheless, the influence of epitaxial stress and strain on the Ga_2_O_3_ phase selection remains significant and cannot be overlooked. Hence, *a*-, *m*-, and *r*-plane-oriented sapphire were subsequently investigated by other authors as a growth substrate for α-Ga_2_O_3_.^30–34^ Nevertheless, due to different growth kinetics across the range of epitaxial techniques reported in the literature, it is not fully clear how sapphire orientation alone affects the assumed phase of Ga_2_O_3_. For instance, in MBE growth on *m*-plane sapphire, it was found that β-Ga_2_O_3_ started to grow on *a*-plane facets of α-(InGa)_2_O_3_ film as the thickness exceeded 50 nm.^32^ In contrast, a recent MOCVD study reported that growth on *m*-plane yields mixed-phase α and β at 100 nm thickness, attributed to β-Ga_2_O_3_ growing on *a*-plane facets on the α-Ga_2_O_3_ layer.^33^ In PLD growth, using the same substrate orientation, phase-pure α-Ga_2_O_3_ films are only possible up to 220 nm before β-Ga_2_O_3_ started to nucleate on *c*-plane facets instead.^34^

In this work, to unambiguously determine the influence of sapphire substrate orientation on Ga_2_O_3_ epitaxy by MOCVD, which is the industry-preferred growth technique, growth must be performed simultaneously on all orientations in the same reactor. The following four sapphire orientations were studied: (112̅0) *a*-plane, (101̅0) *m*-plane, (0001) *c*-plane, and (011̅2) *r*-plane. Systematic investigation of Ga_2_O_3_ crystallographic structure, material quality, and growth mode for each sapphire substrate orientation provides crucial insight toward achieving high-quality α-Ga_2_O_3_ growth by MOCVD.

## Experimental Details

Thin films of Ga_2_O_3_ were simultaneously grown on (11̅20) *a*-plane, (101̅0) *m*-plane, (0001) *c*-plane, and (011̅2) *r*-plane sapphire substrates using an Agnitron Agilis 100 MOCVD reactor. All sapphire substrates were diced into 10 × 7.5 mm^2^ rectangular samples and cleaned using solvents, buffered oxide etch, and a 30 min DI water rinse with ultrasonification before being loaded into the reactor chamber. Triethylgallium (TEGa) and high-purity O_2_ gas (99.9999%) were used as Ga and O precursors with argon (Ar) carrier gas. TEGa flow rate, VI/III ratio, reactor pressure, and growth temperature were held constant at 19.92 μmol/min, 450, 20 Torr, and 500 °C, unless stated otherwise. The surface morphology of the resulting Ga_2_O_3_ layers was characterized using tapping mode atomic force microscopy (AFM), and crystallographic characteristics (crystal structure, quality, and orientation) were studied by X-ray diffraction (XRD) and transmission electron microscopy (TEM). Layer thicknesses were measured by X-ray refractory (XRR) or optical reflectometry. Reflectometry data were fitted using α- and β-Ga_2_O_3_ refractive indices reported by Johnston et al.^35^ All XRD and XRR measurements were performed using a Philips X’pert diffractometer with a Cu Kα source.

## Results and Discussion

Growth time was varied between 2 and 14 min to produce sets of Ga_2_O_3_ films of various thicknesses on the *a*-, *m*-, *c*-, and *r*-plane. Figure 1 shows XRD 2θ-ω scans of all 16 samples grown. In each growth, the thicknesses of the layers were measured to be approximately 13, 20, 50, and 100 nm for all four substrates, i.e., the average growth rate (∼430 nm/h) was found to be independent of crystal orientation. We denote the 16 films as X_1_–X_4_, where X indicates the substrate orientation (A: *a*-plane, M: *m*-plane, C: *c*-plane, and R: *r*-plane), and the subscript corresponds to the film thickness (1:13 nm, 2:20 nm, 3:50 nm, and 4:100 nm). For example, A_3_ refers to the film grown on *a*-plane sapphire with a 50 nm thickness.

**Figure 1:**
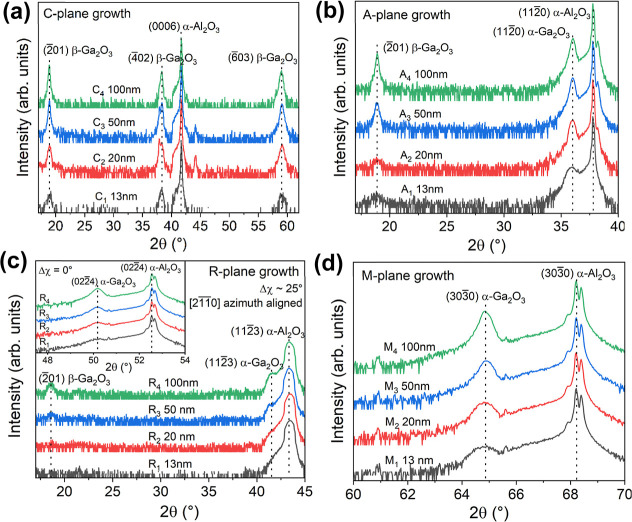
XRD scans of 13–100 nm thick Ga_2_O_3_ films deposited on (a) *c*-plane sapphire, (b) *a*-plane sapphire, (c) *r*-plane sapphire (skew-symmetric geometry with azimuth aligned to [211̅0], tilt ∼25°), and (d) *m*-plane sapphire substrates, under the same growth conditions of *T* = 500 °C, TEGa ∼20 μmol/min, VI/III = 450, and *p* = 20 Torr. The inset of (c) shows XRD scans of the same *r*-plane samples in symmetric geometry. Note that a few spectra show aluminum peaks at 2θ = 38.2°, 44.5°, and 82.3° arising from the diffractometer stage on which samples were directly placed during the scan.

For growth on the *c*-plane (0001) sapphire (Figure 1a), Ga_2_O_3_ nucleated as the β-phase and continued to grow into pure β-Ga_2_O_3_ films with increasing growth time: only {2̅01} β-Ga_2_O_3_ peaks (2θ = 18.9°, 38.3°, and 59.1°) were observed. For growth on *a*-plane sapphire (11̅20), shown in Figure 1b, a mixture of α- and β-Ga_2_O_3_ was detected, as indicated by the presence of both (112̅0) α-Ga_2_O_3_ (2θ = 36.0°) and (2̅01) β-Ga_2_O_3_ peaks in Figure 1b. As the film grows, the intensity of the (2̅01) β-Ga_2_O_3_ peak increases at a faster rate than the (112̅0) α-Ga_2_O_3_ peak—the latter remaining roughly constant as the film thickness exceeded 50 nm, indicating that growth is predominantly β-Ga_2_O_3_ for thicker films.

For layers grown on *r*-plane (011̅2) sapphire, the (022̅4) α-Ga_2_O_3_ peak at 2θ = 50.2° in the symmetric scan (zero tilt) was always present (Figure 1c inset), but from 50 nm thickness onward, the (2̅01) β-Ga_2_O_3_ peak begins to appear in the skew-symmetric scan (25° tilt) aligned to a {112̅3} plane of the sapphire (Figure 1c). Note that the (112̅3) α-Ga_2_O_3_ reflection has a lower relative intensity than the (022̅4) α-Ga_2_O_3_ reflection due to the former’s weaker diffracting power. Likewise, (4̅02) β-Ga_2_O_3_ is not visible in Figure 1c because of a weaker diffracting power than that of (2̅01) β-Ga_2_O_3_, which already has a low intensity. These XRD observations indicate that the film on the *r*-plane begins as predominantly α-Ga_2_O_3_ and becomes a mixed phase at some point during epitaxial growth, likely due to β-Ga_2_O_3_ growing on exposed facets of α-Ga_2_O_3_ growth islands. This can occur via (2̅01) β-Ga_2_O_3_ nucleating on {112̅3} (*n*-plane) facets of α-Ga_2_O_3_, but not exclusively, as later transmission electron microscopy (TEM) analysis revealed the presence of additional orientations of the β-Ga_2_O_3_ crystallite. So far, we have assumed that no bulk α → β transition took place within the initial few monolayers during growth, which is reasonable considering that the growth temperature was fixed at 500 °C—well within the stability limit of α-Ga_2_O_3_.^20^ Such a transformation would also heavily suppress the (011̅2) α-Ga_2_O_3_ peaks, which was not observed. In fact, the (022̅4) α-Ga_2_O_3_ peak increases in intensity as the film grows thicker. Note that since (2̅01) β-Ga_2_O_3_ growing on *n*-plane facets does not result in a detectable peak in symmetric geometry, caution must be exercised when determining the phase composition of Ga_2_O_3_ grown on the *r*-plane. In addition, as previously discussed, a similar growth mode was also observed in MBE growth where the (2̅01) β-Ga_2_O_3_ reflections were found on *c*-plane facets instead.^31^

Finally, only growth on the *m*-plane (101̅0) sapphire resulted in phase-pure α-Ga_2_O_3_ films. The peak attributed to (303̅0) α-Ga_2_O_3_ at 2θ = 64.8° can be seen in Figure 1d. In terms of α-Ga_2_O_3_ crystal quality, the full width at half-maximum (fwhm) of the ω-scan rocking curve was measured to be 0.42° for 50 nm thick layers on the *a*-plane (at (112̅0)), 0.73° for the *m*-plane (at (303̅0)), and 1.18° for the *r*-plane (at (022̅4)).

Investigation of surface morphology (imaged by AFM, Figure 2) of Ga_2_O_3_ grown on *a*-, *m*-, *c*-, and *r*-plane sapphire substrates reveals that from 13 nm layer thickness onward, growth proceeds through 3D islands for *c*-, *a*-, and *r*-plane substrates. On average, the growth island size on *c*- and *a*-planes (24–26 nm wide at 13 nm thickness) is larger than the island size on *m*- and *r*-planes (15–16 nm wide at 13 nm thickness). The increasing size and height of the growth islands manifest as a monotonic increase in surface roughness with film thickness (Figure 3). Notably, the morphological evolution of the film on *c*- and *a*-planes is quite similar, with almost identical surface roughening rates as a function of deposited thickness and island sizes. This correlates with the dominance of β-Ga_2_O_3_ growth on these substrate orientations, especially after 50 nm for the *a*-plane as shown in the XRD results in Figure 1. In contrast, for the *r*-plane, although the growth started with the nucleation of α-Ga_2_O_3_ nanoislands, as the film exceeds 50 nm, large islands began to emerge on the surface of the samples grown on *r*-plane sapphire (Figure 2k); these subsequently grow into elongated islands running along one direction (Figure 2l), resulting in a distinct increase in surface roughness. It is speculated that these elongated islands are a result of β-Ga_2_O_3_ nucleating on inclined facets of α-Ga_2_O_3_ islands as previously discussed, since the emergence of these features coincides with the onset of (2̅01) β-Ga_2_O_3_ peaks in the skew-symmetric XRD scan of the *r*-plane, seen in Figure 1c. Interestingly, at higher growth temperatures only (650 °C), a similar growth mode can be observed on the *m*-plane, which will be discussed below.

**Figure 2:**
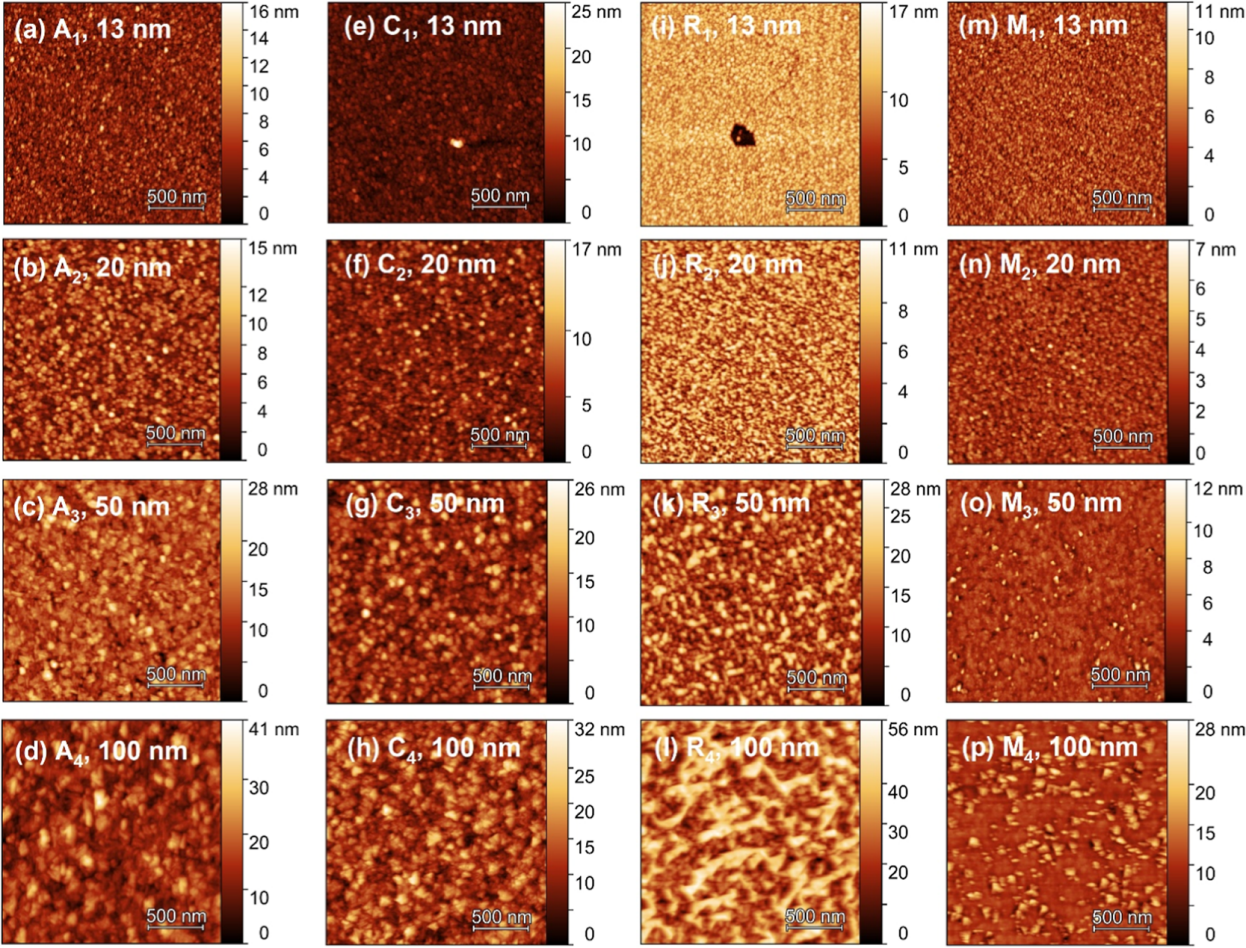
AFM scans (2 × 2 μm^2^ area) of Ga_2_O_3_ films grown on (a–d) *a*-plane (films A_1_–A_4_), (e–h) *c*-plane (films C_1_–C_4_), (i–l) *r*-plane (films R_1_–R_4_), and (m–p) *m*-plane (films M_1_–M_4_) sapphire substrates, with thicknesses between 13 and 100 nm (*T*_gr_ = 500 °C). Note that film names have the format *X*_*i*_, where *X* indicates the substrate orientation (A: *a*-plane, M: *m*-plane, C: *c*-plane, and R: *r*-plane), and the subscript *i* corresponds to the film thickness (1:13 nm, 2:20 nm, 3:50 nm, and 4:100 nm). The surface roughness of all 16 samples is plotted in Figure 3.

**Figure 3:**
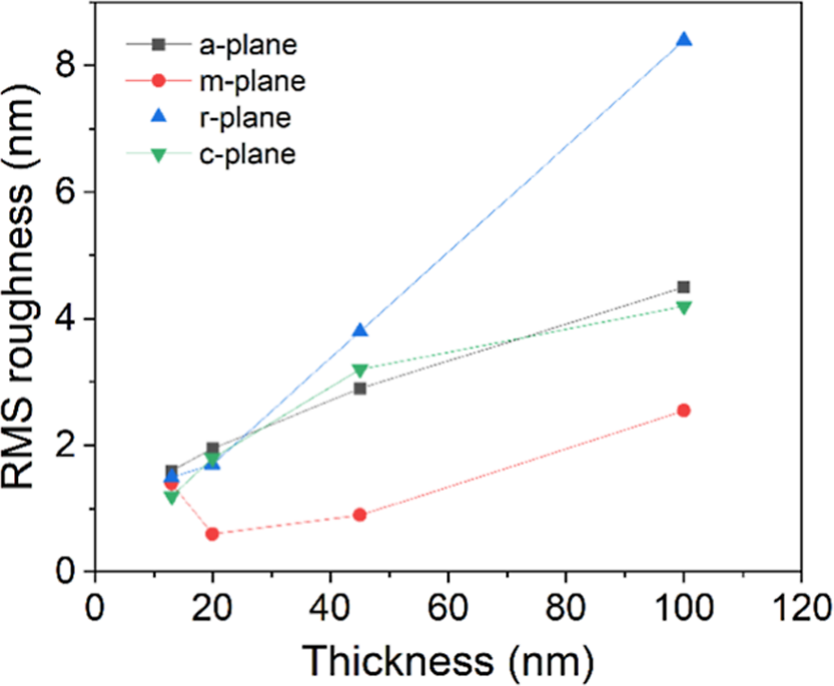
Root-mean-square (RMS) surface roughness of 13–100 nm thick Ga_2_O_3_ films grown on *a*-, *m*-, *c*-, and *r*-planes (*T*_gr_ = 500 °C).

For growth on *m*-plane substrates (Figure 2m–p) at 500 °C, before reaching 20 nm film thickness, it appears that the surface fully coalesces with subnanometer surface roughness. When the epilayer reaches 50 nm thickness, however, new growth hillocks (40–80 nm wide) emerge and are sparsely spaced-out on a very smooth coalesced surface of RMS roughness ∼0.6 nm (Figure 2o), with RMS roughness comparable to the film at a thickness of 20 nm (Figure 2n). These islands subsequently expand to 70–130 nm width (Figure 2p). Hence, it is most likely that the growth mode of α-Ga_2_O_3_ on the *m*-plane is layer + island (Stranski-Krastanov), consistent with observations reported by Li et al.^33^ and Jinno and Okumura.^36^ Compared to the Ga_2_O_3_ films grown at 500 °C discussed so far, Figure 4 shows the XRD scan of *m*-plane Ga_2_O_3_ films grown at a higher growth temperature of 650 °C and their respective AFM scans. As the film grows past 60 nm, XRD 2θ peaks associated with β-Ga_2_O_3_ (2θ = 60.9°) begin to appear near the K_β_ sapphire peak (Figure 4a), coinciding with the emergence of elongated islands running along [0001] in AFM scans (Figure 4c,d). Since similar surface features and XRD peaks have been observed in growth by PLD,^34^ they have been attributed to (2̅01) β-Ga_2_O_3_ nucleating onto *c*-plane facets of α-Ga_2_O_3_ growth islands, which act as nucleation sites for β-Ga_2_O_3_ at higher growth temperatures only.

**Figure 4:**
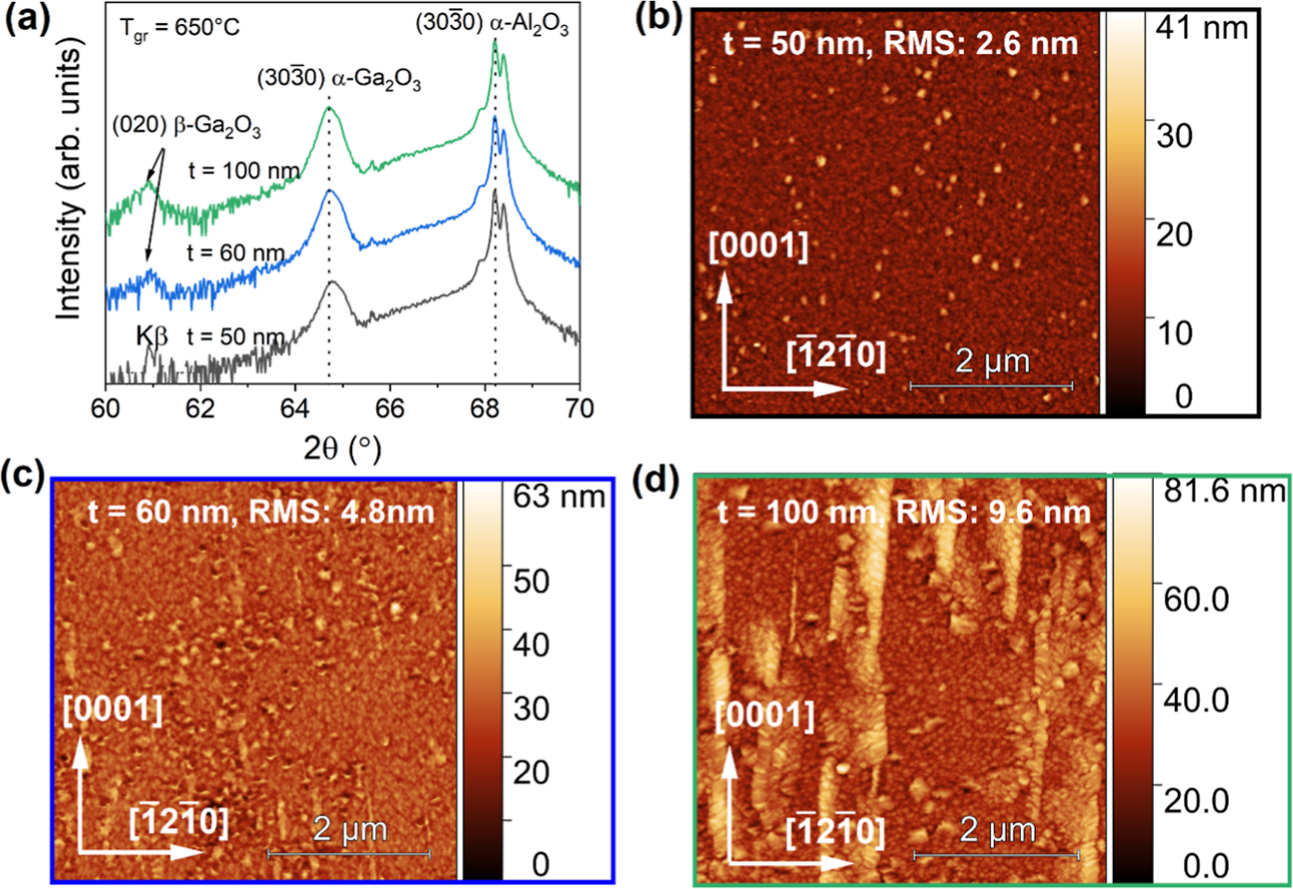
(a) XRD scans of 50–100 nm thick Ga_2_O_3_ films grown on *m*-plane sapphire at 650 °C. AFM scan over a 5 × 5 μm^2^ area of the (b) 50 nm thick, (c) 60 nm thick, and (d) 100 nm thick film.

The in-plane crystallographic relationships among α-Ga_2_O_3_, β-Ga_2_O_3_, and the sapphire were confirmed using XRD skew-symmetric ϕ-scans. In Figure 5a, every α-Ga_2_O_3_ peak on the *a*-, *m*-, and *r*-plane is matched to its sapphire equivalent, signifying one single rotational domain. On the other hand, the six peaks over 360° of β-Ga_2_O_3_ represent six rotational domains. For growth on *c*-plane sapphire, this is the well-known epitaxial relationship <010>(2̅01) β-Ga_2_O_3_ || <101̅0>(0001) α-Al_2_O_3_ as depicted by the model in Figure 5b, which shows similar hexagonal oxygen arrangement for both (0001) α-Al_2_O_3_ and (2̅01) β-Ga_2_O_3_.^37^ However, unlike the *c*-plane, atoms on the *a*-plane of sapphire do not have 3-fold rotation symmetry. Thus, the epitaxial relationship must be slightly different despite the same number of β-Ga_2_O_3_ rotational domains. Noting that β-Ga_2_O_3_ domains are only approximately 60° apart, and that [11̅01] and [11̅01̅] form a 57.6° angle with [11̅00] on either side such that [11̅00] is the bisecting line, the epitaxial relationship is <010>(2̅01) β-Ga_2_O_3_ || [11̅00]/<11̅01>(112̅0) α-Al_2_O_3_ (depicted in Figure 5c). Based on the spacing between neighboring oxygen atoms, the lattice mismatch between β-Ga_2_O_3_ and *a*-plane sapphire is approximately 6.5% for <010> β-Ga_2_O_3_ || [11̅00] α-Al_2_O_3_ and 12.0% for <010> β-Ga_2_O_3_ || <11̅01> α-Al_2_O_3_.

**Figure 5:**
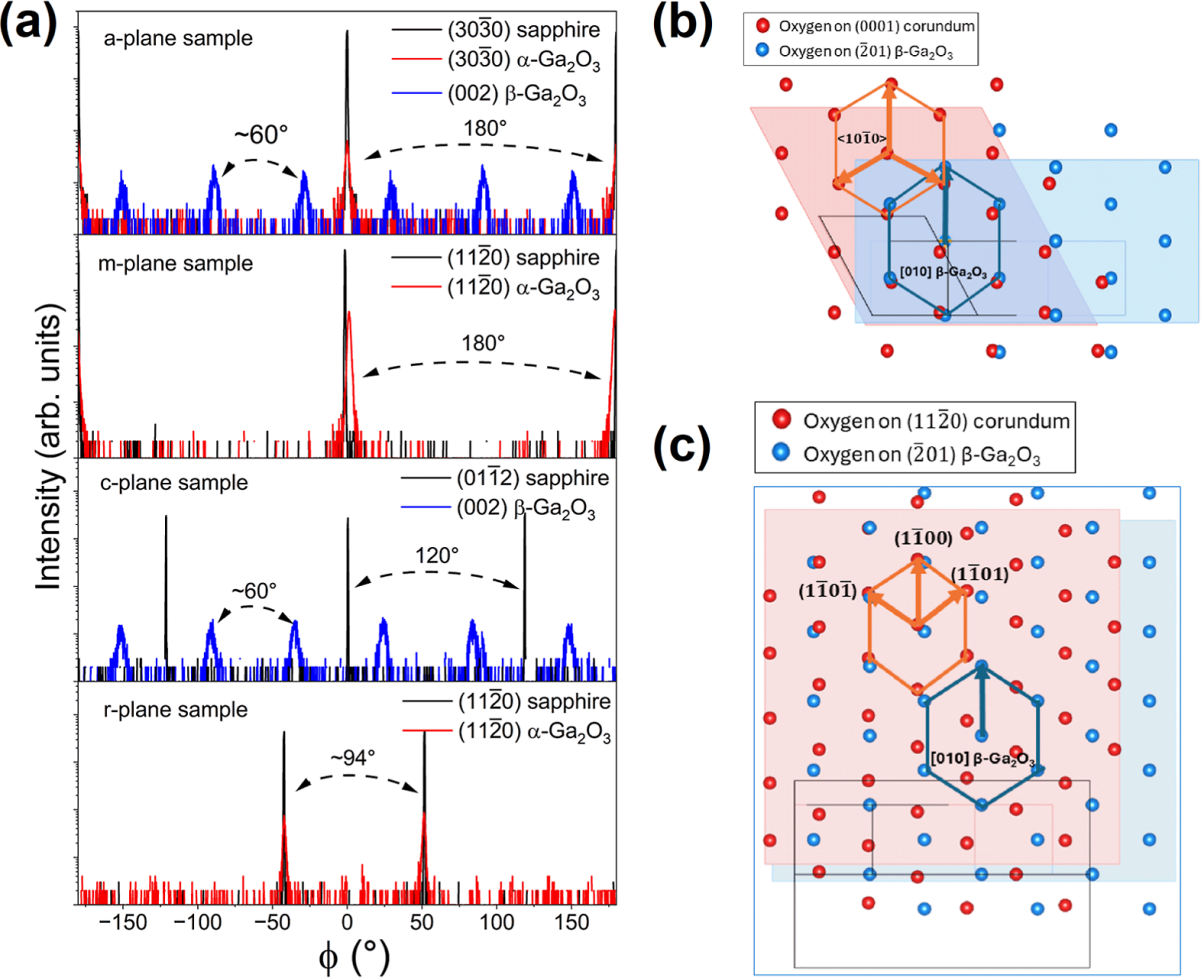
(a) Skew-symmetric ϕ-scans of Ga_2_O_3_ films on *a*-, *m*-, *c*-, and *r*-plane substrates. Visual model of the oxygen atom arrangement of (2̅01) β-Ga_2_O_3_ overlaid on that of (b) *c*-plane sapphire and (c) *a*-plane sapphire. Red spheres represent oxygen atoms on sapphire while blue ones represent oxygen atoms on β-Ga_2_O_3_ (generated with Visualization for Electronic and Structural Analysis (VESTA) software).

Our observations so far suggest that there are two general cases of Ga_2_O_3_ thin film growth on sapphire substrates (Figure 6). In case (a), mixed α- and β- or pure β-grains directly nucleate on the substrate on which there are atoms aligned along <11̅00> α-Al_2_O_3_ (e.g., *c*- and *a*-planes). Eventually, β-grains of Ga_2_O_3_ grow over α-grains, and β-Ga_2_O_3_ becomes the only phase growing. The proposed growth mode of case (a) substrates is consistent with transmission electron microscopy (TEM) observations reported by various authors.^37,38^ In contrast, in case (b), for sapphire planes which do not have atoms aligned along a <11̅00>, such as *m*-plane and *r*-plane substrates, a layer of phase-pure α-Ga_2_O_3_ is stabilized first. However, if growth proceeds via 3D islands, as the α-Ga_2_O_3_ film develops, the β-phase can start nucleating on inclined facets of α-Ga_2_O_3_ islands, resulting in significant surface roughening. This occurs after a certain thickness only, since larger grains provide more suitable facets for β-Ga_2_O_3_ nucleation.^34^ Hence, suppressing island growth and achieving a smooth surface morphology will be crucial to growing phase-pure α-Ga_2_O_3_ on case (b) substrate orientations by MOCVD. For *m*-plane substrates, such a growth mode has been consistently demonstrated across a range of epitaxy methods.^32–34^ However, as previously noted, the specific facet that emerges, which is likely governed by growth kinetics, might vary between different methods.

**Figure 6:**
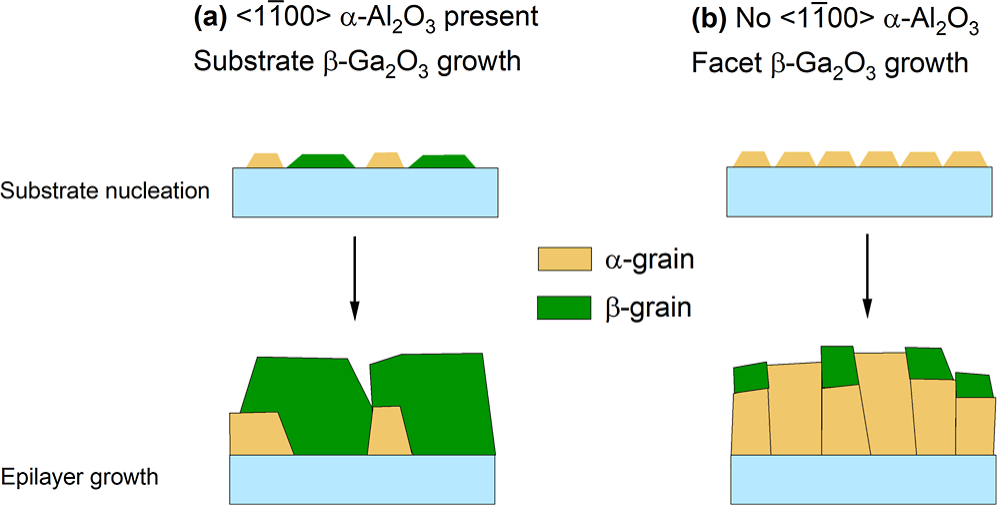
Schematic diagram of the structure evolution of the Ga_2_O_3_ epilayer in two cases: (a) In-plane <11̅00> α-Al_2_O_3_ is present. β-Ga_2_O_3_ can grow directly on the substrate. (b) In-plane <11̅00> α-Al_2_O_3_ is not present. α-Ga_2_O_3_ grows first, but β-Ga_2_O_3_ can nucleate through growth island facets.

To confirm that the structural evolution of Ga_2_O_3_ films grown on *r*-plane substrates is consistent with the proposed growth mode of case (b) substrates, transmission electron microscopy (TEM) images of sample R_3_ (*T*_gr_ = 500 °C, *t* = 50 nm) are shown in Figure 7. A sharp film–substrate interface can be clearly observed in both low- and high-magnification images of Figure 7a,b. The high-resolution TEM (HR-TEM) image of the film in Figure 7b reveals two distinct regions: a lower α-Ga_2_O_3_ region with similar lattice spacing (*d*-spacing) to the substrate and an upper β-Ga_2_O_3_ region with larger *d*-spacing, consistent with the fact that the β-phase has lower density. While the precise α-/β-phase boundary is somewhat indistinct, possibly due to being slightly off edge-on orientation, the β-phase appears to nucleate on top of inclined facets of α-phase crystallites rather than directly on sapphire, appearing as (200) β-Ga_2_O_3_ lattice planes (with a *d*-spacing of approximately 0.6 nm) oriented ∼15° to the (011̅2) α-Ga_2_O_3_ planes in Figure 7b. Note that the dotted white line depicting the boundary in Figure 7b is an approximation since the phase boundary may not be sharply defined: phase overlap and deviation from the line are possible. Local fast Fourier transforms (FFTs) of the substrate and regions where α- and β-phases exist separately further confirm the identity and relative orientation of the crystals. Both α-Ga_2_O_3_ and sapphire show diffraction patterns (DPs) consistent with the [211̅0] zone axis of corundum crystals (Figure 7d,e). The FFT of the β-Ga_2_O_3_ region (Figure 7c) is consistent with the diffraction pattern (DP) of the [010] zone axis. Notably, the Bragg spots of (200) β-Ga_2_O_3_ are misaligned by ∼15° relative to the (011̅2) spots of the corundum crystals, which explains why the β-crystallite observed in Figure 7b is not visible in standard symmetric 2θ-ω scans. Therefore, TEM analysis of the film grown on the *r*-plane substrate shows that, in addition to (2̅01) β-Ga_2_O_3_ growing on (112̅3) α-Ga_2_O_3_ facets found in XRD, β-Ga_2_O_3_ can also grow as (200) β-Ga_2_O_3_ on inclined α-Ga_2_O_3_ facets. Since in the DPs in Figure 7c,d, {202̅} β-Ga_2_O_3_ and {01̅4} α-Ga_2_O_3_ Bragg spots are approximately aligned on the same axis, we speculate that the crystallite consisting of (200) β-Ga_2_O_3_ planes (in Figure 7b) arises from the similar *d*-spacings of {202̅} β-Ga_2_O_3_ planes (*d*-spacing ∼2.8 Å) and {01̅4} α-Ga_2_O_3_ planes (*d*-spacing ∼2.6 Å). Nonetheless, a more detailed TEM investigation will be needed to exhaustively categorize β-phase grains and fully explain their origins on *r*-plane sapphire. Current TEM and XRD analyses collectively support the proposed structural evolution, wherein β-Ga_2_O_3_ nucleates on inclined α-Ga_2_O_3_ facets during growth and not directly on the *r*-plane-oriented substrates (i.e., Figure 6 case (b) substrates).

**Figure 7:**
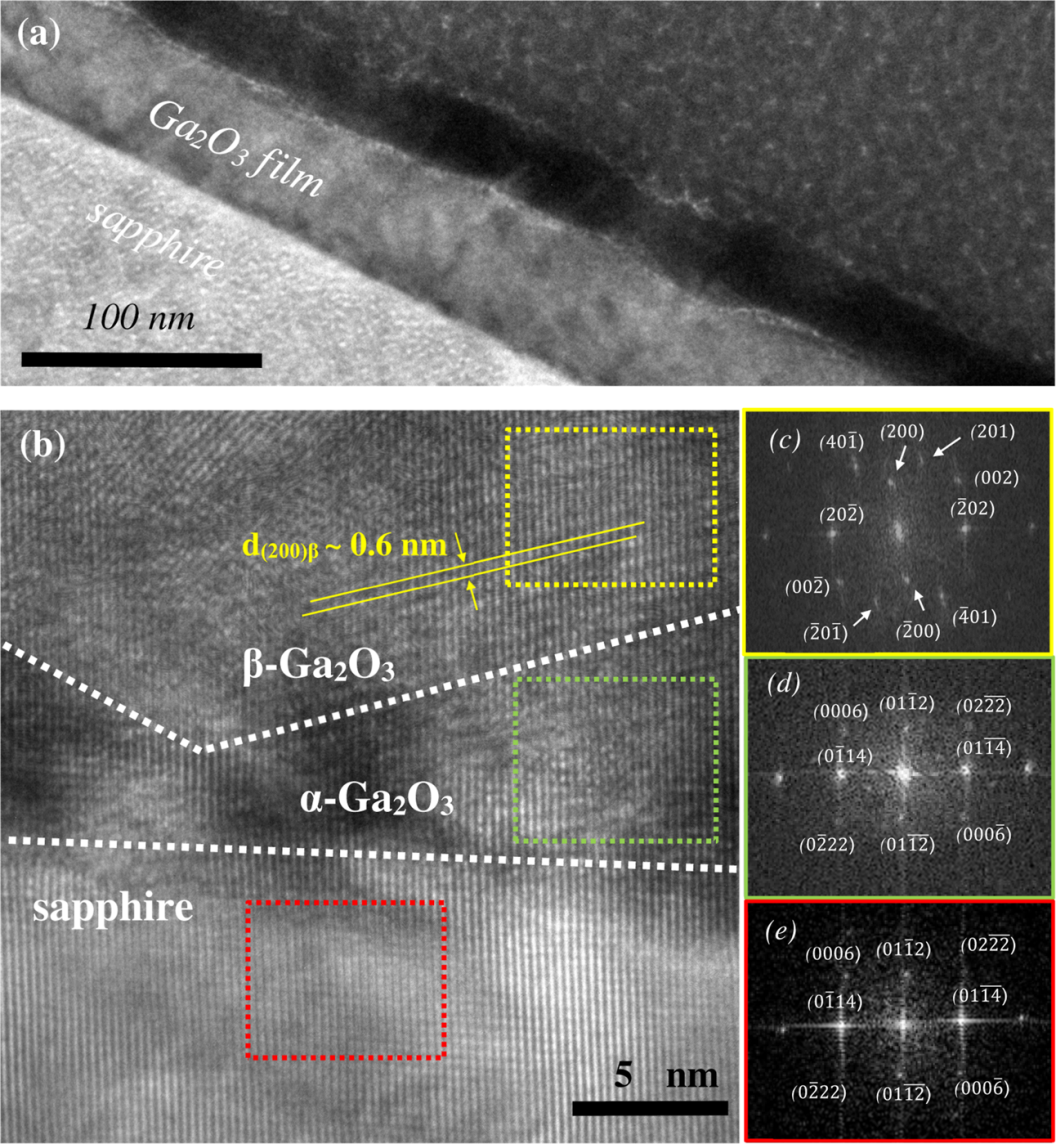
(a) Cross-sectional bright-field (TEM) micrograph of sample R_3_ (*T*_gr_ = 500 °C, *t* = 50 nm). (b) HR-TEM image of a magnified region of sample R_3_. Dotted white lines indicate the approximate locations of α-Ga_2_O_3_/sapphire and α-/β-Ga_2_O_3_ interfaces, inferred by contrast and local diffraction pattern. Fast Fourier transforms (FFT) of selected regions in (b) are shown for (c) β-Ga_2_O_3_ region (dotted yellow square), corresponding to the [010] zone axis; (d) α-Ga_2_O_3_ region (dotted green square), corresponding to the [211̅0] zone axis; and (e) sapphire substrate (dotted red square), corresponding to the [211̅0] zone axis. Reference simulated DPs are provided in Supporting Information.

Overall, under the tested growth conditions thus far, we observed that corundum planes belonging in case (b) tend to favor the growth of α-Ga_2_O_3_, stabilizing at least an initial corundum-phase layer. In particular, the *m*-plane was found to be most conducive to achieving phase-pure α-Ga_2_O_3_ films. In contrast, planes belonging in case (a) tend to have undesirable direct nucleation of β-crystallites. Since growth kinetics were the same on each substrate, the variance in the phase composition evolution of the films must arise chiefly from different strain conditions imposed on the epilayer. Indeed, the growth of α-Ga_2_O_3_ on lattice-matched sapphire is an example of epitaxy-induced phase transformation, in which in-plain epitaxial strain experienced by very thin epilayers modifies the ground state energy such that typically metastable phases, such as α-Ga_2_O_3_, become the most stable.^39^ The key difference between the substrates in case (a) and case (b) appears to be the presence (or lack thereof) of atoms aligned along <11̅00> in the in-plane direction. To qualitatively understand why sapphire substrates with <11̅00> in-plane (i.e., case (a)) often favors the growth of β-Ga_2_O_3_ over that of α-Ga_2_O_3_, let us consider the heterogeneous nucleation of a 3D Ga_2_O_3_ island. Adopting a similar argument outlined by Jesser,^40^ the Gibbs free energy difference Δ*G*_β–α_ between a spherical β- and α-nucleates of the same radius *R* is

1 $$\Delta G_{\beta -\alpha }=\Delta G_{\beta }-\Delta G_{\alpha }=\frac{2}{3}\pi R^{3}\Delta G_{V}+2\pi R^{2}\gamma_{\beta -\alpha }+\pi R^{2}\sigma_{m,\beta -\alpha }+E_{s,\beta -\alpha }$$

The nucleation of α-Ga_2_O_3_ is favored if Δ*G*_β–α_ > 0. The first term of eq 1, which accounts for the difference in condensation energy (per unit volume) Δ*G*_V_ between β- and α-nucleates, is independent of substrate orientation and always negative since β is the most stable phase. The second term accounts for the difference in surface energies γ_β‑α_. Assuming that the four corundum planes considered in this study compete with common monoclinic planes such as (2̅01) and (100), density functional theory calculations suggested that γ_β‑α_ is most likely negative for all four substrates.^41^ The third term accounts for the difference in misfit dislocation energy σ_*m*,β–α_. During the very initial stages of growth, this term is negligible because islands can be fully strained. The last term, which accounts for the difference in strain energy *E*_s,β–α_, is the only positive term since α-Ga_2_O_3_ is lattice-matched to sapphire. Hence, if the magnitude of *E*_s,β–α_ is greater than that of $\frac{2}{3}\pi R^{3}\Delta G_{V}+2\pi R^{2}\gamma_{\beta -\alpha }$ , α-Ga_2_O_3_ growth should be initially favored. Past studies have estimated that, if the nearest-neighbor mismatch between the stable phase (β-Ga_2_O_3_) and the substrate (α-Al_2_O_3_) is much larger (greater than 10–20%) compared to the mismatch between the metastable phase (α-Ga_2_O_3_) and the substrate (ideally as close to zero as possible), then growth islands forming on the substrate prefer to assume the metastable phase.^39,40^ However, for sapphire orientations in which the in-plane epitaxial relationship <010> β-Ga_2_O_3_ || <101̅0> α-Al_2_O_3_ is possible, such as *c*-plane, *a*-plane, or *n*-plane, the mismatch between α-Ga_2_O_3_ and sapphire is comparable to the mismatch between β-Ga_2_O_3_ and sapphire. Based on the arrangement of oxygen atoms, the distance between nearest neighboring atoms along [11̅00] is 3.02 Å for α-Ga_2_O_3_ and 2.86 Å for α-Al_2_O_3_, resulting in a mismatch of 5.6%. In comparison, the mismatch of <010> β-Ga_2_O_3_ || < 11̅00> α-Al_2_O_3_ is 6.5%. This is far from the ideal case considered in theory. As such, to minimize free energy, growth islands prefer to assume the β-phase: the energy cost of inducing more in-plane strain in the system is lower than the cost of assuming the α-phase.

Nonetheless, according to eq 1, it is possible to enhance the nucleation of the metastable α-Ga_2_O_3_ over β for case (a) substrates. Solving for *R* by setting $\frac{\partial \left(\Delta G_{\beta -\alpha }\right)}{\partial R}=0$ gives a constant critical radius R* above which growth islands will exclusively prefer the stable phase over the metastable phase.^40^ This signifies that, to successfully grow metastable polymorphs, the island size must be kept below R*. In MOCVD growth, the island size can be reduced by increasing Ga supersaturation via a combination of higher TEGa flow rate and lower VI/III ratio. This theory was tested for *a*-plane and *c*-plane substrates using a TEGa flow rate of ∼23 μmol/min (15% increase from 20 μmol/min), a VI/III ratio of 200 (125% reduction from 450), and a total pressure of 10 Torr (halved from 20 Torr) while maintaining the growth temperature at 500 °C. Note that pressure was reduced here to minimize the probability of homogeneous β-Ga_2_O_3_ nucleation from the gas phase,^42^ which can occur under high TEGa flow, despite higher chamber pressure increasing supersaturation. The XRD 2θ-ω scan of the resulting 70 nm thick Ga_2_O_3_ film on *a*-plane sapphire (Figure 8a) shows peaks arising from α-Ga_2_O_3_ and the substrate only without any {2̅01} β-Ga_2_O_3_ peaks. In terms of crystallinity, ω-scan rocking curves measured at the (112̅0) α-Ga_2_O_3_ peak show excellent quality, achieving a fwhm of 62 arcsec, comparable to α-Ga_2_O_3_ grown on the *c*-plane by mist-CVD.^1,2^ This marked improvement is owed to the film being phase-pure α-Ga_2_O_3_, unlike the mixed-phase film obtained using the initial growth conditions. However, phase-pure α-Ga_2_O_3_ growth on a *c*-plane substrate still proves challenging. Under the same conditions for which phase-pure α-Ga_2_O_3_ was grown on *a*-plane sapphire, the film grown on the *c*-plane is mixed-phase β and κ-Ga_2_O_3_ instead. In the XRD 2θ-ω scan in Figure 8b, a new set of peaks appear to the right of the {2̅01} β-Ga_2_O_3_ peaks, which can be attributed to κ-Ga_2_O_3_. It is difficult to distinguish the first set of {2̅01} β-Ga_2_O_3_ and {002}­κ-Ga_2_O_3_ peaks around 2θ = 19°, but the separation is more noticeable toward higher 2θ angles, for example, (6̅03) β-Ga_2_O_3_ at 2θ = 59.1° versus (006) κ-Ga_2_O_3_ at 2θ = 59.8°. Nonetheless, our results demonstrate that increasing TEGa flow rate and decreasing VI/III ratio (with preferably low chamber pressure) can favor the growth of metastable Ga_2_O_3_ (α- or κ-) on substrates with <11̅00> α-Al_2_O_3_ present (tested for *c*- and *a*-plane substrates), in agreement with nucleation theory of metastable polymorphs. In addition, our observations for growth on *c*-plane substrates are consistent with a previous growth MOCVD study,^43^ in which κ-Ga_2_O_3_ was stabilized on *c*-plane sapphire by lowering VI/III ratios, and similar diffraction patterns were observed. Interestingly, pushing growth conditions into metal-rich regimes (i.e., low VI/III ratio for MOCVD) seems to favor the growth metastable polymorphs of Ga_2_O_3_ (α- or κ-) on sapphire substrates with <11̅00> α-Al_2_O_3_ for MBE^32,44^ and PLD^45^ as well.

**Figure 8:**
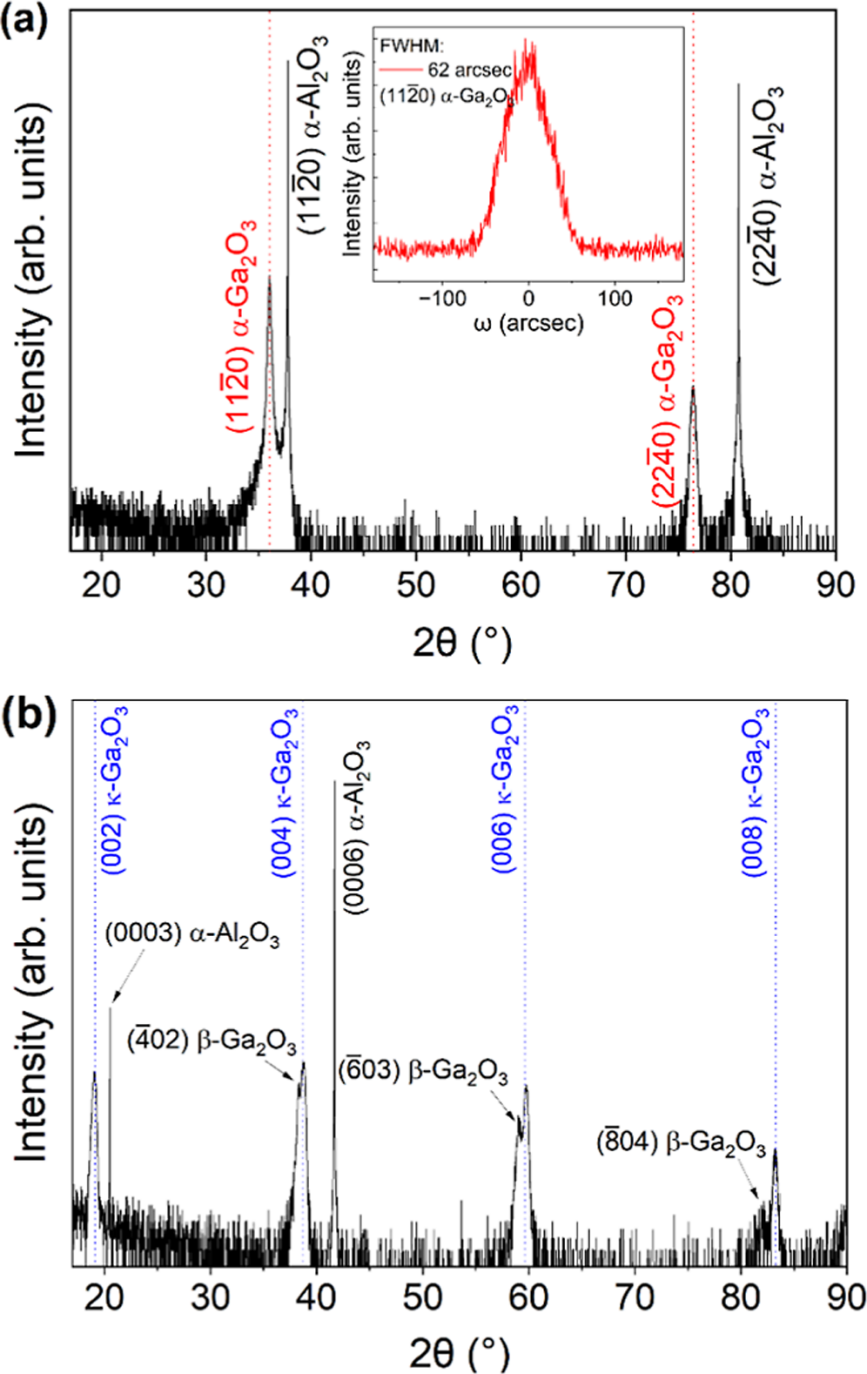
XRD scans of Ga_2_O_3_ films grown on (a) *a*-plane sapphire (with the inset showing the ω-scan rocking curve of the (112̅0) α-Ga_2_O_3_ peak) and (b) *c*-plane sapphire under the same growth conditions of *T* = 500 °C, TEGa∼ 23 μmol/min, VI/III = 200, and *p* = 10 Torr. A Ge(220) 4-bounce monochromator was used to obtain these scans.

## Conclusions

By performing simultaneous growth of Ga_2_O_3_ using MOCVD, on *a*-, *m*-, *c*-, and *r*-plane sapphire substrates, and systematically characterizing the resulting Ga_2_O_3_ films by XRD and AFM, we have shown that the structural evolution of the Ga_2_O_3_ film depends on the presence of <11̅00> α-Al_2_O_3_ in the substrate. For substrate orientations with <11̅00> α-Al_2_O_3_, such as *c*- and *a*-planes, β-Ga_2_O_3_ tends to directly nucleate at the start of growth, making the film either phase-pure β-Ga_2_O_3_ or mixed-phase α- and β-Ga_2_O_3_. On the other hand, for substrate orientations without <11̅00> α-Al_2_O_3_, such as *m*- and *r*-planes, α-Ga_2_O_3_ tends to nucleate first without any sizable β-Ga_2_O_3_ grains present, but 3D island growth needs to be suppressed to prevent the emergence of facets that act as nucleation sites for β-crystallites. Such a growth mode was directly observed for growth on *r*-plane substrates via TEM. Despite differences in the growth mechanism, the growth rate of the Ga_2_O_3_ was found to be mostly independent of the substrate orientation. Finally, classical nucleation theory was adopted to gain insight on the phase selectivity and help identify favorable conditions for α-Ga_2_O_3_ epitaxy on sapphire substrates with <11̅00> α-Al_2_O_3_. As a result, we have demonstrated phase-pure α-Ga_2_O_3_ (with 62 arcsec rocking curve fwhm) can be grown on *a*-plane substrates by MOCVD under conditions with higher Ga supersaturation, but for *c*-plane substrates so far, only mixed-phase β- and κ-Ga_2_O_3_ films could be obtained. These results serve as a valuable foundation for optimizing scalable α-Ga_2_O_3_ growth toward future device fabrication.

## Supplementary Material



## Notes

Simulated DPs of the [010] zone of β-Ga_2_O_3_ and the [211̅0] zone of corundum crystals (PDF)
