# Supplementary material for: Heteroepitaxial Growth of α‑Ga2O3 by MOCVD on a‑, m‑, r‑, and c‑Planes of Sapphire
Source: Cryst Growth Des. 2025 Jul 29;25(16):6529–38. doi: 10.1021/acs.cgd.5c00183 (PMC12372763; doi:10.1021/acs.cgd.5c00183)
Supplement: Supplementary file 1 [file cg5c00183_si_001.pdf]

Supporting Information

Heteroepitaxial growth of α-Ga_2O_3 by MOCVD on a-, m-, r- and c-plane of sapphire

*Khai D. Ngo, Indraneel Sanyal, Matthew D. Smith, and Martin Kuball**

Centre for Device Thermography and Reliability, University of Bristol, Bristol, United Kingdom

E-mail: Martin.Kuball@bristol.ac.uk

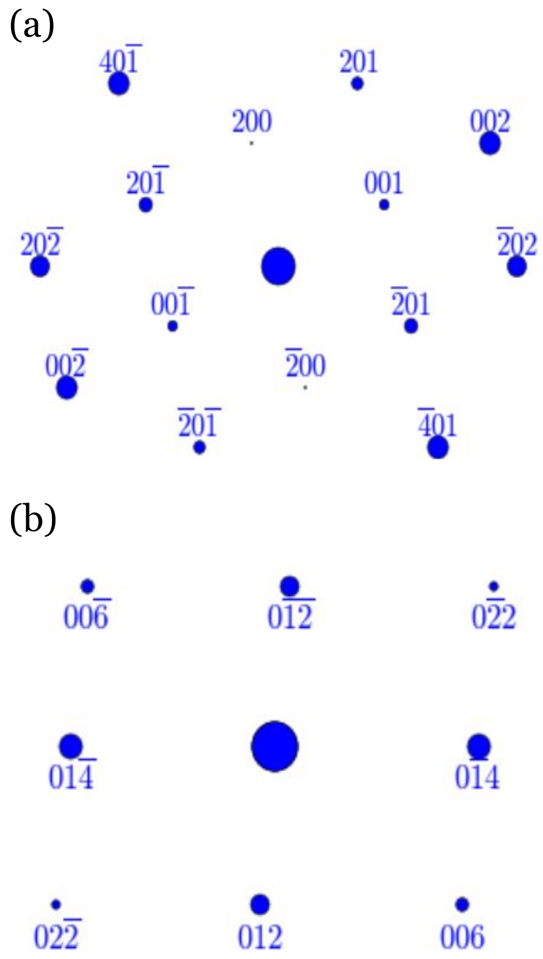

Figure S1: Simulated diffraction patterns (obtained using PTCLab) of (a) [010] zone of β-Ga_2O_3, and (b) $[2\bar{1}\bar{1}0]$ zone of corundum crystals.
